# High COX‐2 expression in cancer‐associated fibiroblasts contributes to poor survival and promotes migration and invasiveness in nasopharyngeal carcinoma

**DOI:** 10.1002/mc.23150

**Published:** 2019-12-22

**Authors:** Yinghong Zhu, Chen Shi, Liang Zeng, Guizhu Liu, Weihong Jiang, Xin Zhang, Shilian Chen, Jiaojiao Guo, Xingxing Jian, Jian Ouyang, Jiliang Xia, Chunmei Kuang, Songqing Fan, Xuan Wu, Yangbowen Wu, Wen Zhou, Yongjun Guan

**Affiliations:** ^1^ Department of Otolaryngology Head and Neck Surgery, Xiangya Hospital Central South University Changsha China; ^2^ Key Laboratory for Carcinogenesis and Invasion, Chinese Ministry of Education, Key Laboratory of Carcinogenesis, Chinese Ministry of Health, Cancer Research Institute and School of Basic Medical Science Central South University Changsha China; ^3^ Department of Oncology Second Affiliated Hospital of Soochow University Suzhou China; ^4^ Department of Pathology Guangzhou Women and Children's Medical Center Guangzhou China; ^5^ Key Laboratory of Nutrition and Metabolism, Institute for Nutritional Sciences Chinese Academy of Sciences Shanghai China; ^6^ Shanghai Center for Bioinformation Technology Shanghai Academy of Science and Technology Shanghai China; ^7^ Department of Pathology Second Xiangya Hospital of Central South University Changsha China

**Keywords:** cancer‐associated fibroblasts, COX‐2, metastasis, nasopharyngeal carcinoma

## Abstract

Nasopharyngeal carcinoma (NPC) has the highest rate of metastasis among head and neck cancers, and distant metastasis is the major reason for treatment failure. We have previously shown that high cyclooxygenase‐2 (COX‐2) expression is associated with a poor prognosis of patients with NPC and inhibits chemotherapy‐induced senescence in NPC cells. In this study, we found that COX‐2 was upregulated in cancer‐associated fibroblasts (CAFs) derived from NPC by RNA‐Seq. Furthermore, elevated COX‐2 expression in CAF was detected in NPC patients with poor survival and distant metastasis by using immunohistochemistry. Then, we identified that COX‐2 is highly expressed in CAF at the distant metastasis site in seven paired NPC patients. High expression of COX‐2 and secretion of prostaglandin E2, a major product catalyzed by COX‐2 in fibroblasts, promotes migration and invasiveness of NPC cells in vitro. On the contrary, inhibition of COX‐2 has the opposite effect in vitro as well as in the COX‐2^−/−^ mouse with the lung metastasis model in vivo. Mechanistically, we discovered that COX‐2 elevates tumor necrosis factor‐α expression in CAF to promote NPC cell migration and invasiveness. Overall, our results identified a novel target in CAF promoting NPC metastasis. Our findings suggested that high expression of COX‐2 in CAF may serve as a new prognostic indicator for NPC metastasis and provide the possibility of targeting CAF for treating advanced NPC.

## 1 INTRODUCTION

Nasopharyngeal carcinoma (NPC) is a type of head and neck cancer that exhibits an endemic distribution with a high prevalence in Southern China and Southeast Asia.^1^, ^2^ The etiologic factors for NPC include Epstein–Barr virus (EBV) infection, ethnics, genetic susceptibility, and environmental factors, including consumption of food with volatile nitrosamines.^3^, ^4^, ^5^ Upon diagnosis, most patients present with metastasis to the regional lymph nodes or even distant organs. The common sites of distant metastasis of NPC are the bone, lung, liver, and retroperitoneal lymph nodes.^6^, ^7^, ^8^ Most distant metastasis occurs within 3 years after radiotherapy completion, with distant metastasis occurring in 52% of patients in the 1st year, 23% in the 2nd year, and 20% in the 3rd year.^8^ To date, although NPC is sensitive to radiotherapy, distant metastasis is the primary cause of treatment failure.^9^

Tumor metastasis is closely related to tumor microenvironment (TME). The TME has cellular components and noncellular extracellular matrix (ECM).^10^, ^11^, ^12^ There are evidence suggesting that EBV‐infected NPC cells interacted with TME components to facilitate metastasis. An increased presence of Foxp3^+^ Treg cells and CD68^+^ tumor‐associated macrophages (TAMs) has been found in EBV‐positive NPC specimens and associated with poor prognosis.^13^ Another recent study has revealed an interacting loop between NPC cells and TAMs in driving NPC metastasis. In plethora of tumor microenvironmental cell types, cancer‐associated fibroblasts (CAFs) have involved as one of the most promising targets owing to their abundant presence and functional significance in various tumor entities, including multiple myeloma, oral cancer, and gastric cancer.^14^, ^15^, ^16^ CAF can be phenotypically identified based on markers such as fibroblast activation protein α (FAP), α‐smooth muscle actin (SMA), and FSP‐1.^12^, ^17^, ^18^, ^19^ Several studies have shown that CAF can be used as an important prognostic factor in a variety of tumors.^20^, ^21^, ^22^ Chen^23^ reported that overexpression of α‐SMA‐positive fibroblasts (CAFs) in NPC predicts poor prognosis. As a major and important component of tumor matrices, CAF plays an important role in tumor invasiveness and metastasis. CAF can also promote dissemination and metastasis through engaging in heterotypic interactions with tumor cells in ovarian cancer.^24^ Considering CAF is a major component in TME of NPC, however, its clinical significance in the invasiveness and metastasis of NPC has rarely been reported.

Cyclooxygenase (COX) is a rate‐limiting enzyme in prostaglandin biosynthesis. There are two isoforms of COX—COX‐1 and COX‐2. COX‐1 is constitutively expressed in a number of tissues and mainly plays a role in tissue homeostasis. By contrast, COX‐2 is an inducible enzyme responsible for the production of prostaglandins at sites of inflammation and wound‐healing.^25^ Of note, COX‐2 is highly expressed in numerous types of human cancer, such as breast, ovarian, colorectal cancer, and NPC.^26^, ^27^, ^28^, ^29^ Our previous studies reported that COX‐2 serves as a marker of poor prognosis in NPC, and COX‐2 expression induces proliferation and chemoresistance of NPC cells.^30^ However, stromal expression of COX‐2 has not been specifically evaluated in NPC to date.

In this study, we first found that COX‐2 is highly expressed in CAF from patients with NPC by RNA‐seq analysis. Subsequently, we observed clinical significance of the expression of COX‐2 in CAF correlated with lymph‐node (N) stage, metastasis (M) stage, relapse, and survival in patients with NPC. Further functional study of COX‐2 in CAF will be explored on NPC metastasis in vitro and in vivo with CAF derived from primary NPC patients, NPC cell lines, and COX‐2 knockout (COX‐2^−/−^) mouse model. Our goal in this study is to identify a reliable, clinically useful prognostic marker for predicting NPC metastasis and offer a novel clinical opportunities for treating advanced NPC.

## 2 MATERIALS AND METHODS

### 2.1 Cell culture and regents

Human NPC cell lines including CNE1 and CNE2, mouse lung cancer cell line LLC, were cultured in high‐glucose Dulbecco's modified Eagle's Medium (DMEM; Invitrogen, Carlsbad, CA) supplemented with 10% fetal bovine serum (FBS; Gibco, Grand Island, NY), 100 units/mL of penicillin, and 100 μg/mL of streptomycin (P/S). WI38, the human lung normal fibroblast cell, was kindly provided by Dr. Yu Sun (Shanghai Institute of Nutrition and Health, Chinese Academy of Sciences, China).

Human normal fibroblast (NF) and CAF were derived from opposite normal nasopharynx and nasopharyngeal carcinoma tissues from patients with newly diagnosed NPC. Fibroblasts isolation procedure was as described previously.^31^ Briefly, the fresh tissues were cut into pieces and isolated using Type I collagenase digestion for 30 minutes at 37°C and were thereafter cultured in low‐glucose DMEM medium for about 1 week until formation of fibroblasts. Specimens were obtained with written informed consent from patients with NPC enrolled in the Xiangya Hospital of Central South University (CSU). The NF and CAF used for further functional studies were less than third passages.

The following reagents were used in this study: NS398 (a selective COX‐2 inhibitor) and prostaglandin E2 (PGE2) were purchased from Cayman Chemicals (Cayman, MI). The recombinant human protein tumor necrosis factor‐α (TNF‐α) and TNF‐α neutralizing antibody were purchased from Sino Biological (SB Inc, Beijing, China).

Human anti‐COX‐2 antibody was purchased from Cell Signaling (Cell Signaling Technology, MA) and human anti‐α‐SMA antibody was purchased from Abcam (Abcam, MA). Anti‐β‐actin, anti‐GAPDH antibodies, and horseradish peroxidase (HRP)‐conjugated IgG secondary antibodies were from Santa Cruz Biotechnology (Santa Cruz, CA), and anti‐TNF‐α was from ABclonal (ABclonal Biotech Co., Hubei, China).

### 2.2 Conditioned medium derived from human normal fibroblast and cancer‐associated fibroblasts

NF and CAF (2 × 10^5^/mL), mouse fibroblast (2 × 10^5^/mL), and WI38 cells (2 × 10^5^/mL) were plated into six‐well culture plates in low‐glucose DMEM supplemented with 10% FBS and cultured overnight, and subsequently refreshed with 0.5 mL of serum‐free high‐glucose DMEM, and then 2 mL of serum‐free high‐glucose DMEM was added to six‐well culture plates. The culture supernatants were harvested after 48 hours. Then, cell debris was removed with centrifugation and stored at −80°C until experimentation. To obtain conditioned medium (CM) from fibroblast cells with NS398 or PGE2, CAF derived from patients with NPC, WI38‐COX‐2Ctr cells, and fibroblasts from COX‐2^+/+^ mice were cultured in serum‐free high‐glucose DMEM with 20 μM NS398, or NF derived from patients with NPC, WI38‐COX‐2sh cells and fibroblasts from COX‐2^−/−^ mice were cultured in serum‐free high‐glucose DMEM with 10 μM PGE2. The CM from these cultures was assayed for wound‐healing assay and transwell of NPC cells as described below.

### 2.3 Patients and clinical samples

Two primary samples of NPC patients for RNA‐Seq were obtained from Xiangya Hospital, CSU (Changsha, China). Serum from healthy donor (HD) (n = 14), primary NPC patients (n = 18), and NPC patients with metastasis (n = 14) for detection of PGE2 were from Xiangya Hospital. Paraffin‐embedded nontumor (NT, n = 11), NPC (n = 43), and the paired NPC with primary site and distant metastasis site (n = 7) for immunohistochemistry (IHC) analysis were collected from Tumor Hospital, CSU (Changsha, China) after informed consent from patients.

All studies with human samples were approved by Medical Ethics Committee of CSU. Clinical characteristics for NPC patients are summarized in Table 1.

**Table 1:** The correlation of COX‐2 expression in CAF and clinical characteristics in NPC

Cases (n = 43)		COX‐2 expression in CAF		*P*‐value
		High (n = 16)	Low (n = 27)	
Sex				.089
Male	27 (62.7%)	8	19	
Female	16 (37.3%)	8	8	
Age, years				.051
<50	25 (58.1%)	11	14	
>50	18 (41.9%)	5	13	
T stage (tumor extent)				.058
T1+T2	29 (67.4%)	9	20	
T3+T4	14 (32.6%)	7	7	
N stage (lymph note involvement)				.001**
N0	6 (14%)	0	6	
N1+N2+N3	37 (86%)	16	21	
NPC clinical stage				.012*
I+II	16 (37.3%)	4	12	
III+IV	27 (62.7%)	12	15	
Relapse				.02*
No	28 (63%)	7	21	
Yes	15 (37%)	9	6	
Death				.034*
No	26 (60.4%)	6	20	
Yes	17 (39.6%)	10	7	

*Note*: T stage (tumor extent): T1: nasopharynx, oropharynx, and nasal fossa, T2: parapharyngeal extension, adjacent soft tissue involvement (medial pterygoid, lateral pterygoid, prevertebral muscles), T3: bony structure involvement (skull base, cervical vertebra, paranasal sinuses), T4: intracranial extension, cranial nerve, hypopharynx, orbit, extensive soft tissue involvement (beyond the lateral surface of the lateral pterygoid muscle, parotid gland); N stage (lymph note involvement): N0: none, N1: Retropharyngeal (regardless of laterality), cervical: unilateral, ≤6 cm, and above caudal border of cricoid cartilage, N2: cervical: bilateral, ≤6 cm, and above caudal border of cricoid cartilage, N3: >6 cm and/or below caudal border of cricoid cartilage (regardless of laterality); NPC clinical stage: I: T1N0M0, II: T2N0‐1M0, T1N1M0, III: T3N0‐2M0, T1‐2N2M0, IV: T4N0‐3M0, T1‐3N3M0, T1‐4N0‐3M1; COX‐2: cyclooxygenase‐2

Abbreviations: CAF, cancer‐associated fibroblast; COX‐2, cyclooxygenase‐2; NPC, nasopharyngeal carcinoma.

* Indicates a statistically significant difference (*P *< .05) as determined by the *χ* ^2^ test.

** Indicates a statistically significant difference (*P *< .01) as determined by the *χ* ^2^ test.

### 2.4 Immunocytochemistry analysis

IHC was performed for detecting the expression of COX‐2, α‐SMA, and TNF‐α on paraffin‐embedded slides. The detailed procedure was performed as previously described.^32^ In brief, the slides were subjected to dewaxing, rehydration, and hydrogen‐peroxide treatment. Subsequently, the tissue sections were incubated with anti‐COX‐2, α‐SMA, and TNF‐α antibodies in 1:1000, 1:1000, and 1:500 dilution overnight at 4°C. On the next day, the slides were incubated with HRP‐conjugated secondary antibody and stained with 3,3′‐diaminobenzidine tetrahydrochloride hydrate for 3 minutes. Finally, cell nuclei were counterstained with hematoxylin. Staining was observed under a microscope, as described previously. Briefly, semiquantitative assessment of COX‐2, α‐SMA, and TNF‐α immunostaining was performed by calculating both intensity of staining (0, 1, 2, or 3) and extent of staining (0: 0%; 1: <10%; 2: 10%‐50%; 3: >50%).^33^ The stained sections were evaluated and scored independently by two pathologists who were blinded to clinical parameters.

### 2.5 Enzyme‐linked immunosorbent assay

To measure PGE2 release, serum samples were obtained from peripheral blood, CM was collected 48 hours after fibroblasts or WI38 cells were cultured in serum‐free high‐glucose DMEM. PGE2 levels in the supernatants were measured by using PGE2 enzyme‐linked immunosorbent assay (ELISA) kits (Cayman Chemicals, MI), respectively, according to the manufacturer's instructions. Standard curves were processed in parallel for individual experiments to achieve precise quantification of sample concentrations.

### 2.6 Reverse‐transcriptase polymerase chain reaction

Total RNA was isolated from fibroblast cells and WI38 cells with TRIzol reagent (Invitrogen Life Technologies, CA) as instructed by manufacturer's protocol. Complementary DNAs were then synthesized from 2 μg of total RNA with SuperScript III First‐Strand Synthesis SuperMix (Thermo Fisher Scientific, MA) according to the manufacturer's protocol. Amplification of specific targets was performed to determine the messenger RNA (mRNA) levels using Bio‐Rad iCycler iQ Real‐Time PCR Detection System (Bio‐Rad, CA). Glyceraldehyde 3‐phosphate dehydrogenase (GAPDH) was used as a loading control. The specific primers used for amplification are summarized in Table 2. The relative mRNA levels were calculated as the value of $2^{{\Delta C}_{t}}$ normalized to the control.

**Table 2:** Summary of primer sequences, annealing temperature, and PCR product sizes for 44 target genes

Primer	Reference sequence number	Sequence (5′→3′)	Temperature (°C)	Product size (bp)
COX‐2/PTGS2	NM_000963.3	AGTCCCTGAGCATCTACGGTTTG	62.25	180
		CCTATCAGTATTAGCCTGCTTGTCT	59.99	
EP1	NM_000955.2	TATCATGGTGGTGTCGTGCAT	59.79	154
		GATGTACACCCAAGGGTCCAG	60.07	
EP2	NM_000956.3	TCTGCTCCTTGCCTTTCACG	60.60	173
		ACAACAGAGGACTGAACGCAT	59.93	
EP3	NM_198715.2	GGCCATTCAGCTTATGGGGA	59.81	127
		CTGCTTCTCCGTGTGTGTCT	59.97	
EP4	NM_000958.2	CTGGTGGTGCTCATCTGCTC	60.74	136
		GGATGGGGTTCACAGAAGCA	59.96	
mPGES‐1	NM_004878.4	CAGTATTGCAGGAGCGACCC	60.81	96
		GACGAAGCCCAGGAAAAGGA	59.96	
15‐PGDH	NM_000860.5	TTGGAAGACTGGACATTTTGG	56.34	145
		CCTTCACCTCCATTTTGCTT	56.20	
αSMA/ACTA2	NM_001141945.2	TAGCACCCAGCACCATGAAG	60.04	104
		CTGCTGGAAGGTGGACAGAG	60.04	
FAP	NM_004460.3	GCTCTGGTTAATGCACAAGTGG	60.10	109
		GGAAGTGGGTCATGTGGGTG	60.61	
PDGFRα	NM_006206.4	CTGCCTGACATTGACCCTGT	59.96	98
		GAACCCGTCTCAATGGCACT	60.32	
PDGFRβ	NM_002609.3	CCGTCCTCTATACTGCCGTG	59.69	163
		CAGGAGATGGTTGAGGAGGTG	59.79	
FSP‐1/S100A4	NM_019554.2	GGGCAAAGAGGGTGACAAGT	60.18	142
		GTCCCTGTTGCTGTCCAAGT	60.18	
tenascin‐C/TN‐C	NM_002160.3	TCACCAACTGTGCTCTGTCC	59.89	138
		TTGAGTGTTCGTGGCCCTTC	60.53	
cytokeratin 8/CK8	NM_001256282.1	GAAGACCACCAGCGGCTAT	59.48	187
		AGACACCAGCTTCCCATCAC	59.67	
CD31	NM_000442.4	TGCCGTGGAAAGCAGATACT	59.39	162
		GGAGCAGGGCAGGTTCATAA	59.74	
NG2/CSPG4	NM_001897.4	TTGCTGTGGCTGTGTCTTTTG	59.87	170
		ATCATGCTCTGAGCGCTGG	60.23	
podoplanin/PDPN	NM_006474.4	CATCGGCTTCATTGGTGCAA	59.47	165
		CACGGGTCATCTTCTCCCAC	60.11	
CXCL5/ENA‐78	NM_002994.4	AAGGTGGAAGTGGTAGCCTC	59.02	206
		CCTTCTTGTCTTCCCTGGGT	58.93	
CXCL12/SDF‐1	NM_000609.6	GAGCCAACGTCAAGCATCTC	59.28	113
		CCACTTTAGCTTCGGGTCAAT	58.29	
CXCL16	NM_022059.3	TGGCACCTGACTCTAATACCT	57.88	200
		CAGTGGCTGGTTAGTCCTATGTT	60.06	
CXCL1/GROα	NM_001511.3	GAAAGCTTGCCTCAATCCTG	57.06	107
		CACCAGTGAGCTTCCTCCTC	59.75	
CXCL3/GROg	NM_002090.2	AACCGAAGTCATAGCCACAC	57.91	104
		TGCTCCCCTTGTTCAGTATC	56.92	
CXCL11/I‐TAC	NM_005409.4	CAGTTGTTCAAGGCTTCCCC	59.04	201
		GCCTTGCTTGCTTCGATTTG	58.66	
CCL22	NM_002990.4	ACAGACTGCACTCCTGGTTG	59.89	111
		ACGTAATCACGGCAGCAGAC	60.74	
CCL2/MCP‐1	NM_002982.3	AAGAATCACCAGCAGCAAGT	57.72	166
		CTTGGGTTGTGGAGTGAG	54.84	
CCL8/MCP‐2	NM_005623.2	TTCTGTGCCTGCTGCTCATG	60.96	155
		TTGGATGTTGGTGATTCTTGTGTAG	59.53	
CCL13/MCP‐4	NM_005408.2	AAGTCTCTGCAGTGCTTCTGT	59.58	156
		TGATCACATAGCTCTTCAGCC	57.53	
CCL20/MIP3α	NM_004591.2	GTGTGCGCAAATCCAAAACA	58.43	148
		AAACCTCCAACCCCAGCAAG	60.47	
CCL11/Eotaxin	NM_002986.2	CCCCAGAAAGCTGTGATCTTCA	60.29	113
		GGAGTTGGAGATTTTTGGTCCAGAT	61.04	
CCL25/TECK	NM_005624.3	CCATCAGCAGCAGTAAGAGG	58.05	131
		CTGTAGGGCGACGGTTTTAT	57.70	
RANTES/CCL5	NM_002985.2	CAGTCGTCTTTGTCACCCGA	59.97	236
		TGTAACTGCTGCTGTGTGGT	59.82	
IL‐1β	NM_000576.2	GTACCTGTCCTGCGTGTTGA	59.97	153
		GGGAACTGGGCAGACTCAAA	59.89	
IL‐6	NM_000600.4	AGCCACTCACCTCTTCAGAAC	59.65	83
		ACATGTCTCCTTTCTCAGGGC	59.72	
IL‐8/CXCL8	NM_000584.3	TACTCCAAACCTTTCCACCC	57.04	158
		AACTTCTCCACAACCCTCTG	57.06	
IL‐7	NM_000880.3	CCAGTTGCGGTCATCATGACTA	60.42	113
		TGATGCTACTGGCAACAGAACA	60.22	
IFN‐α	NM_024013.2	CCAGTTCCAGAAGGCTCCAG	60.04	178
		CCTCTCCTCCTGCATCACAC	59.82	
TNF‐α	NM_000594.3	ACCTCTCTCTAATCAGCCCTCT	59.48	91
		GGGTTTGCTACAACATGGGCTA	60.88	
IL‐5	NM_000879.2	GCAAGGGGGTACTGTGGAAA	59.89	195
		TTTGGCTGCAACAAACCAGT	59.10	
HGF	NM_000601.5	GGACAAGAACATGGAAGACT	54.98	163
		ACAACGAGAAATAGGGCAAT	55.03	
Epiregulin/EREG	NM_001432.2	CGTGTGGCTCAAGTGTCAAT	58.77	171
		GCTTAAAGGTTGGTGGACGG	59.12	
VEGFA	NM_001025366.2	CGAAGTGGTGAAGTTCATGGATG	59.87	238
		TATGTGCTGGCCTTGGTGAG	60.04	
IGF1	NM_001111283.2	GCTCTTCAGTTCGTGTGTGG	59.14	99
		ATCCACGATGCCTGTCTGAG	59.54	
IGF2	NM_000612.5	GACACCCTCCAGTTCGTCT	58.65	99
		ACAGCACTCCTCAACGATGC	60.67	
GAPDH	NM_001289746.1	TCGGAGTCAACGGATTTGGT	59.32	154
		TGGAATTTGCCATGGGTGGA	59.88	

Abbreviations: COX‐2, cyclooxygenase‐2; GAPDH, glyceraldehyde 3‐phosphate dehydrogenase; IL‐6, interleukin 6; PCR, polymerase chain reaction; SMA, smooth muscle actin; TNF‐α, tumor necrosis factor‐α.

### 2.7 Western blot analysis

Western blot was performed as previously described.^34^, ^35^ CAF derived from patients with NPC and NPC cells treated with CM were lysed in radioimmunoprecipitation assay buffer containing protease inhibitor cocktail and phosphatase inhibitor cocktails (Roche, Basel, Switzerland) on ice, and the protein levels were quantified by using BCA protein assay kit (Dingguo Biotech Co., Beijing, China), according to the manufacturer's instruction. The total protein was separated with 10 to 12% sodium dodecyl sulfate‐polyacrylamide gel electrophoresis and analyzed by Western blot analysis.

### 2.8 Immunofluorescence

Cells were seeded onto glass coverslips with an appropriate confluent overnight, and were washed with phosphate‐buffered saline (PBS) and fixed with 4% paraformaldehyde, permeabilized with 0.5% Triton X‐100, and blocked with 4% bovine serum albumin. Subsequently, the samples were incubated with primary antibodies for α‐SMA (1:100), COX‐2 (1:500), E‐cadherin (1:100), and Vimentin (1:500) overnight at 4°C, followed by incubation with Alexa fluor‐488‐conjugated secondary antibody or Alexa fluor‐594‐conjugated secondary antibody. To stain the nuclei, 4′,6‐diamidino‐2‐phenylindole (DAPI) was used, and samples were photographed under a fluorescence microscope.

### 2.9 Establishment of stable COX‐2 short‐hairpin RNA knockdown cell lines

GV248 lentiviral vectors with a GFP label containing short‐hairpin RNA (shRNA) targeting COX‐2 (AACTGCTCAACACCGGAATTT) and a scramble sequence (TTCTCCGAACGTGTCACGT) as a control were purchased from Genechem (Genechem Co., Shanghai, China). WI38 was kindly provided by Prof. Yu Sun (Department of Cardiology, the First Affiliated Hospital of Sun Yat‐Sen University), the WI38 cells was transfected with these constructs using Lipofectamine® 3000 transfection reagent (Invitrogen). Cells were selected using 1 μg/mL puromycin for 2 weeks, and stable cell lines were obtained.

### 2.10 Wound‐healing assay

CM was collected as described above. For NPC cell treatment, CM was diluted with equivalent serum‐free medium. NPC cells were cultured with complete medium in six‐well plates at a density of 5 × 10^5^ cells per well and incubated for 100% confluency. Then, to examine cell invasiveness, we scratched with a 200‐μL tip, and washed with PBS three times. Finally, NPC cells were cultured with CM at 48 hours and invasiveness was detected at 0, 12, 18, 24, and 48 hours, respectively.

### 2.11 Transwell migration assay

NPC cells were resuspended (2 × 10^4^ cells/well) in 200 μL of serum‐free medium, and placed in the upper compartment of a transwell chamber without Matrigel. The lower compartment was filled with 500 μL of CM and 10% FBS. After 24 hours of incubation, NPC cells penetrated through the membrane were fixed with methanol and stained with 0.05% Crystal Violet. Three random fields of cells were counted in each.

### 2.12 Tumor metastasis assay in COX‐2 knockout mouse model

All animal experiments were performed in accordance with the guidelines of the Institutional Animal Care and local Veterinary Office and Ethics Committee of the Central South University (CSU). COX‐2^−/−^ was kindly provided by Dr. Ying Yu (Institute for Nutritional Sciences, Shanghai, China). LLC was purchased from PerkinElmer (PerkinElmer Inc). LLC cells (10^6^ cells in 200 μL PBS) were injected into COX‐2 wild type (COX‐2^+/+^) mice (n = 5) and COX‐2^−/−^ mice (n = 4) at 10‐weeks old through tail vein. When COX‐2^+/+^ mice exhibited asthma and thin after 4 weeks of observation, all animals were then killed. Lung tissues were dissected from mice for hematoxylin‐eosin staining and IHC.

### 2.13 Statistical analysis

Analyses were performed using GraphPad Prism 7 software (GraphPad Prism Inc, CA). The two‐tailed *t*‐test was utilized for the comparison of two conditions. The statistical tests were analyzed as paired where appropriate. *P* < .05 was considered statistically significant and marked as *P* < .05*, *P* < .01**, and *P* < .001***. Graphical results are presented as mean ± standard error of the mean (SEM).

## 3 RESULTS

### 3.1 COX‐2 was upregulated in CAF and correlates with metastasis in nasopharyngeal carcinoma

**Figure 1:**
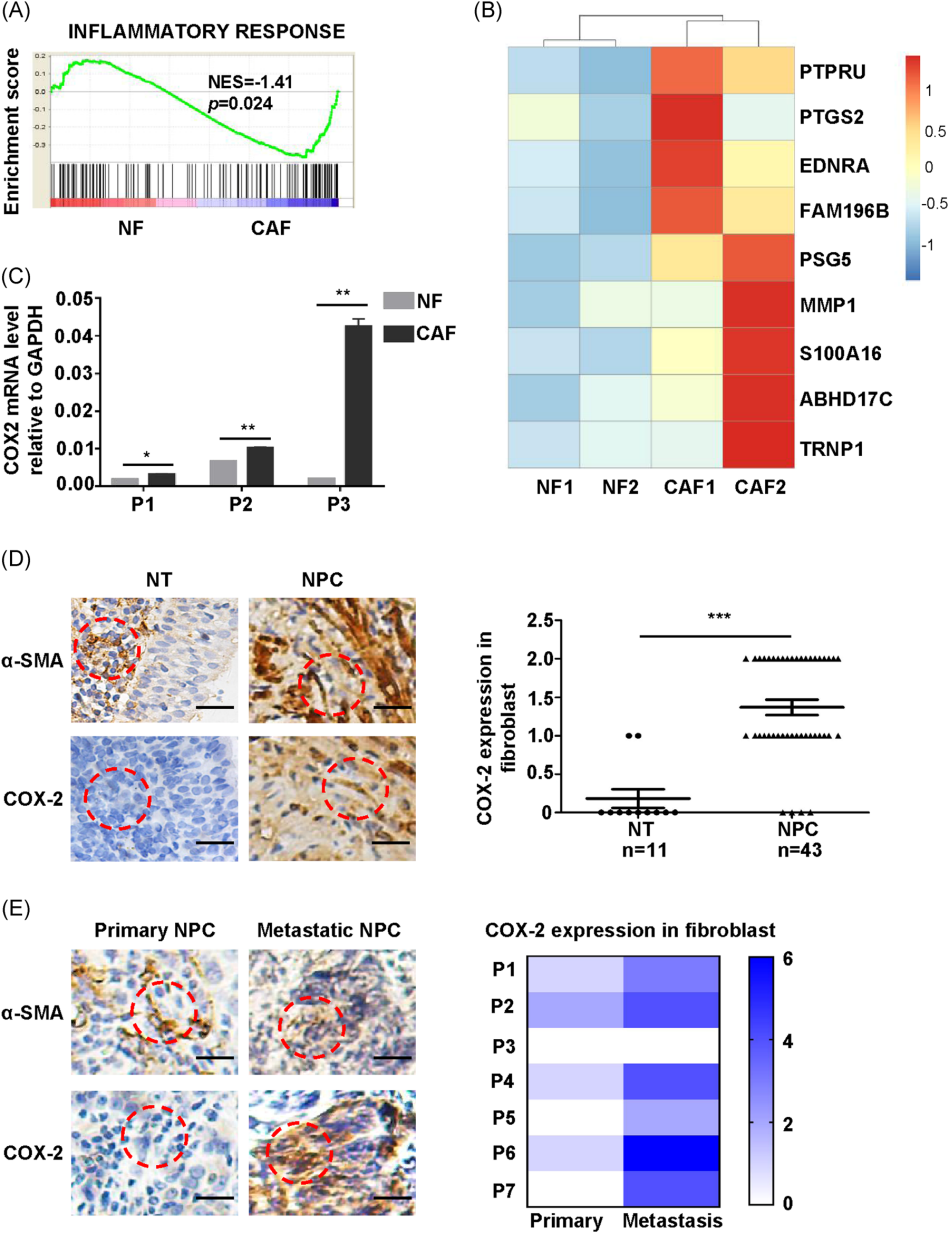
Cyclooxygenase‐2 (COX‐2) was upregulated in cancer‐associated fibroblast (CAF) and correlates with metastasis in nasopharyngeal carcinoma (NPC). A, Gene Set Enrichment Analysis (GSEA) of inflammatory response‐related genes in paired normal fibroblast (NF) and CAF. B, Heatmap of nine differentially expressed genes between NF and CAF (Log_2_ (FC) > 1.2) by RNA‐seq analysis. C, Assessment COX‐2 messenger RNA (mRNA) expression in paired NF and CAF from three patients by quantitative polymerase chain reaction (q‐PCR). Bar, standard error of the mean (SEM). *P < .05, **P < .01 by unpaired *t* test. D, Left, representative images of α‐smooth muscle actin (α‐SMA) and COX‐2 immunohistochemistry (IHC) staining in NT and NPC. Scale bars, 20 μm. Right, statistical chart represents the COX‐2 score in fibroblast (NT= 11, NPC= 43). Column, mean; bar, SEM. ***P < .001 by unpaired *t* test. E, Left, representative images of α‐SMA and COX‐2 IHC staining in paired patients at primary site and distant metastasis site. Scale bars, 20 μm. Right, heatmap represents the COX‐2 score in fibroblast (n = 7). **P < .01 by paired *t* test [Color figure can be viewed at wileyonlinelibrary.com]

Paired NF and CAF (n = 2) derived from patients with NPC were stained with α‐SMA, an established marker of fibroblasts (Figure S1A). RNA‐sequencing was applied to examine the critical gene expression between primary NF and CAF. We discovered that the major type of gene signature is the inflammatory response pathway (Figure 1A), among which PTGS2 is ranked at second (Figure 1B). Interestingly, PTGS2, also known as COX‐2, a key molecule in inflammatory response, was the most upregulated gene in CAF compared with NF.

We next verified whether COX‐2 was upregulated in CAF by quantitative reverse‐transcriptase polymerase chain reaction (qRT‐PCR). The mRNA levels of COX‐2 were indeed elevated in CAF compared with NF in three paired NPC patients (Figure 1C). Then, we examined the expression of COX‐2 on protein level in CAF indicated as α‐SMA‐positive cells in NPC. IHC staining also revealed a marked increase of COX‐2 expression in CAF compared with those from NT (Figure 1D). Furthermore, the expression of COX‐2 in CAF was examined by IHC in 43 patients with NPC, among which 16 patients with NPC were identified as high expression of COX‐2, the others were low expression of COX‐2. Then, the correlation between COX‐2 expression in CAF and clinical characteristics of NPC was investigated. As a result, the expression of COX‐2 in CAF was not significantly correlated with age (*P* = .089), gender (*P* = .051), and T stage (*P* = .058), but was positively correlated with N stage (*P* = .001), NPC clinical stage (*P* = .012). N stage and NPC clinical stage are identified by tissue involvement, thus COX‐2 in CAF may be involved in metastasis in NPC (Table 1). In addition, we also showed that a high expression of COX‐2 in CAF was positively correlated with relapse (*P* = .02) and poor survival (*P* = .034) in patients with NPC, suggesting that the high expression of COX‐2 in CAF confers poor outcome in NPC (Table 1). To further confirm the relevance of COX‐2 and metastasis in NPC, seven paired NPC sequential samples with primary site and distant metastasis site that include two lung metastasis (P5 and P6) and five cervical lymph‐node metastasis (P1, P2, P3, P4, and P7) were explored to examine the COX‐2 expression in CAF. Interestingly, we found that COX‐2 was significantly upregulated in CAF derived from distant metastasis sites compared with primary sites (Figure 1E). Taken together, these results indicate that altered COX‐2 levels in CAF may promote metastasis in NPC.

### 3.2 Increased COX‐2 and PGE2 secretion from CAF promotes migration of NPC cell lines

**Figure 2:**
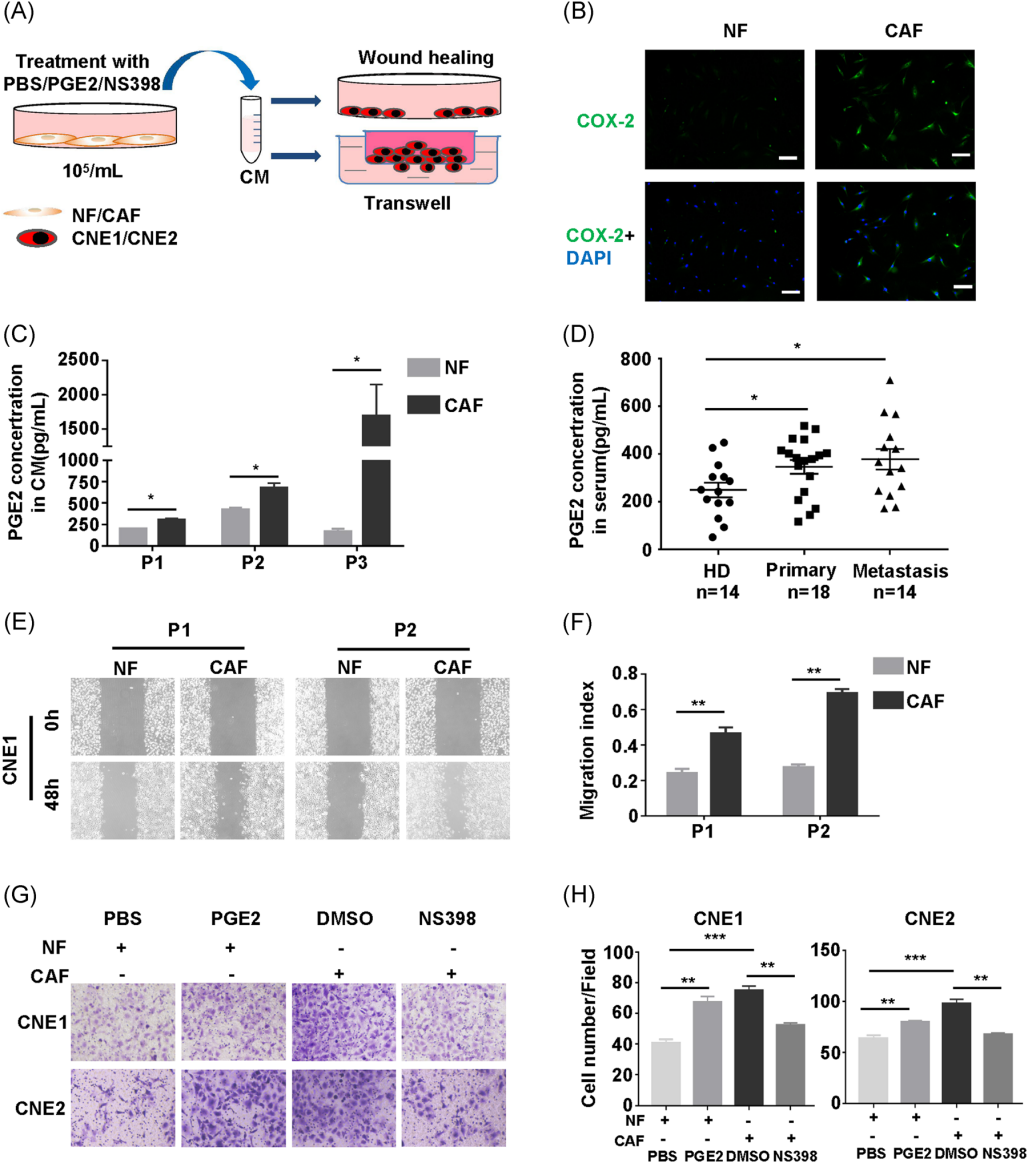
Increased COX‐2 and prostaglandin E2 (PGE2) secretion from CAF promotes migration of NPC cell lines. A, Schematic diagram for the treatment of NPC cells with conditioned medium (CM) collected from NF and CAF. B, Representative images of COX‐2 expression in NF and CAF by IF. 4′,6‐diamidino‐2‐phenylindole (DAPI), blue; COX‐2, green. Scale bars, 10 μm. C, Assessment PGE2 concentration in CM from NF and CAF (n = 3) by enzyme‐linked immunosorbent assay (ELISA) analysis. Bar, SEM. *P < .05 by unpaired *t* test. D, Assessment PGE2 levels in serum from healthy donor (HD; n = 14), primary NPC (n = 18), and metastatic NPC (n = 14) by ELISA analysis. Bar, SEM. *P < .05 by the Student *t* test. E, Representative images of the migration of CNE1 treated with CM from NF and CAF at 0 and 48 hours by the wound‐healing assay. F, Migration index analysis of CNE1 treated with CM from NF and CAF. Bar, SEM. **P < .01 by unpaired *t* test. G, Representative images of the invasiveness of CNE1 and CNE2 treated with CM from NF and CAF by transwell. H, Histograms represent the number of invaded cells. Bar, SEM. **P < .01, ***P < .001 by unpaired *t* test. CAF, cancer‐associated fibroblast; COX‐2, cyclooxygenase‐2; NF, normal fibroblast; NPC, nasopharyngeal carcinoma; SEM, standard error of the mean [Color figure can be viewed at wileyonlinelibrary.com]

Schematic diagram for the treatment of NPC cells with CM collected from NF and CAF (Figure 2A). To detect the function of COX‐2 in CAF, first, we applied IF and found that COX‐2 was upregulated in CAF (Figure 2B). Then, considering that PGE2 is the major product catalyzed by COX‐2, we tested PGE2 level in CM from three paired NF and CAF by ELISA. Consistent with COX‐2 expression, CAF secreted more PGE2 than NF (Figure 2C). Importantly, we confirmed the presence of elevated PGE2 levels in serum from 18 primary NPC patients, especially in the 14 NPC with metastatic group compared with 14 healthy donors by using ELISA (Figure 2D).

Considering the relevance of PGE2 in NPC patients with metastasis, cell migration and invasiveness assays were applied to examine the migration and invasiveness capacities of COX‐2 in NPC cells in vitro. First, CNE1 was treated by CM from NF and CAF, we found migration index of CNE1 was higher in the CAF group (Figure 2E,F) by wound‐healing assay. Meanwhile, we also revealed CM from CAF contributed to invasiveness of CNE1 and CNE2 (Figure 2G) by invasiveness assay. Consistently, exogenous PGE2 was used to mimic COX‐2 overexpression in NF. The invasiveness of CNE1 and CNE2 was enhanced when incubated with CM from NF with PGE2 treatment compared with CM from NF alone (Figure 2H). In contrast, opposite trends were found in NPC cells cultured with CM from CAF with NS398, a selective COX‐2 inhibitor, treatment, compared with CM from CAF alone (Figure 2H). To characterize the epithelial to mesenchymal transition features of NPC cells during migration and invasiveness, we also found higher expression of Vimentin and lower expression of E‐cadherin in CNE1 treatment with CM from CAF by Immunofluorescence (IF) (Figure S2). These data suggest that COX‐2/PGE2 in CAF results in enhanced migration and invasiveness of NPC cells in vitro.

### 3.3 The fibroblasts from COX‐2 knockout mice attenuates migration and invasiveness of NPC cells in vitro

**Figure 3:**
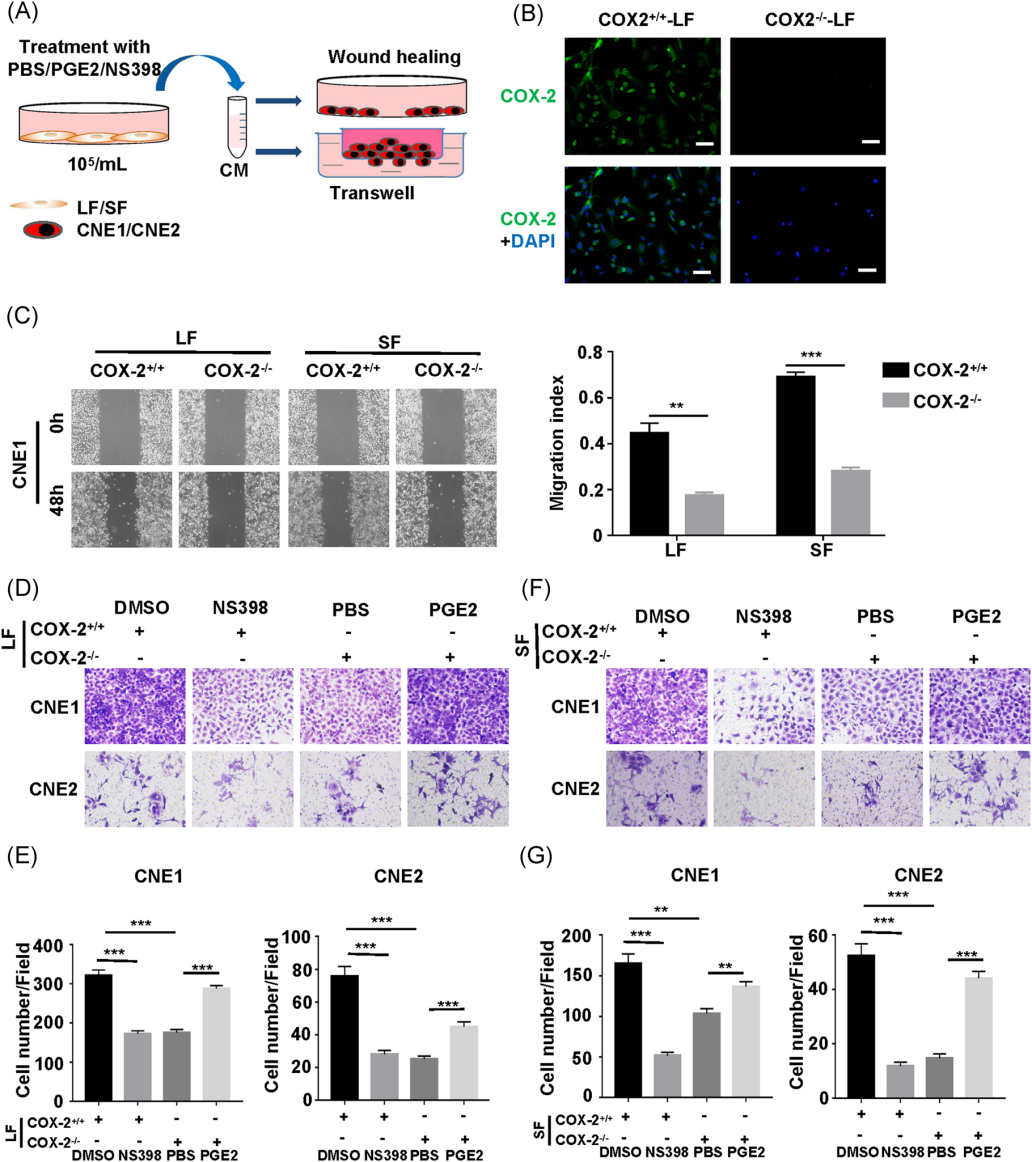
The fibroblasts from COX‐2 knockout mice attenuates migration and invasiveness of NPC cells in vitro. A, Schematic diagram for the treatment of NPC cells with CM collected from LF and SF. B, Representative images of COX‐2 expression in COX‐2^+/+^‐LF and COX‐2^−/−^‐LF by IF. DAPI, blue; COX‐2, green. Scale bars, 10 μm. C, Left, representative images of the migration of CNE1 treated with CM from LF and SF at 0 and 48 hours by wound‐healing assay. Right, migration index analysis of CNE1 treated with LF CM and SF CM. Bar, SEM. **P < .01, ***P < .001 by unpaired *t* test. D, Representative images of the migration of CNE1 and CNE2 treated with CM from LF by transwell. E, Histograms represent the number of migrate cells. Bar, SEM. ***P < .001 by unpaired *t* test. F, Representative images of the migration of CNE1 and CNE2 treated with CM from SF by transwell. G, Histograms represent the number of migrate cells. Bar, SEM. **P < .01, ***P < .001 by unpaired *t* test. CM, conditioned medium; COX‐2, cyclooxygenase‐2; DAPI, 4′,6‐diamidino‐2‐phenylindole; NPC, nasopharyngeal carcinoma; SEM, standard error of the mean [Color figure can be viewed at wileyonlinelibrary.com]

Schematic diagram for the treatment of NPC cells with CM collected from LF and SF (Figure 3A). To further validate the functional role of COX‐2 in fibroblasts, we generated fibroblasts derived from skin (SF) and lung (LF) from COX‐2^+/+^ (COX‐2^+/+^‐SF and COX‐2^+/+^‐LF) and COX‐2^−/−^ (COX‐2^−/−^‐SF and COX‐2^−/−^‐LF) mice, respectively. As expected, the expression of α‐SMA in SF and LF was detected by IF (Figure S3A) and COX‐2 protein was hardly detectable in COX‐2^−/−^‐SF and COX‐2^−/−^‐LF (Figures 3B and S3B). We subsequently examined whether CM from COX‐2^−/−^‐SF and COX‐2^−/−^‐LF affects migration and invasiveness of CNE1 and CNE2. We found that CNE1 and CNE2 treated with CM from COX‐2^−/−^‐SF and COX‐2^−/−^‐LF significantly have impaired migration abilities compared with treated by CM from COX‐2^+/+^‐SF and COX‐2^+/+^‐LF (Figure 3C), we also found that CNE1 and CNE2 treated with CM from COX‐2^−/−^‐SF and COX‐2^−/−^‐LF significantly have impaired invasiveness phenotypes compared with treated by CM from COX‐2^+/+^‐LF (Figure 3D,E) and COX‐2^+/+^‐SF (Figure 3F,G). While PGE2 treatment reversed the inhibitory effect of CM from COX‐2^−/−^‐LF (Figure 3D,E) and COX‐2^−/−^‐SF (Figure 3F,G), on the contrary, NS398 treatment reversed the enhanced effect of CM from COX‐2^+/+^‐LF (Figure 3D,E) and COX‐2^+/+^‐SF (Figure 3F,G).

### 3.4 The COX‐2‐knockdown in WI38 cells attenuates migration and invasiveness of NPC cells

**Figure 4:**
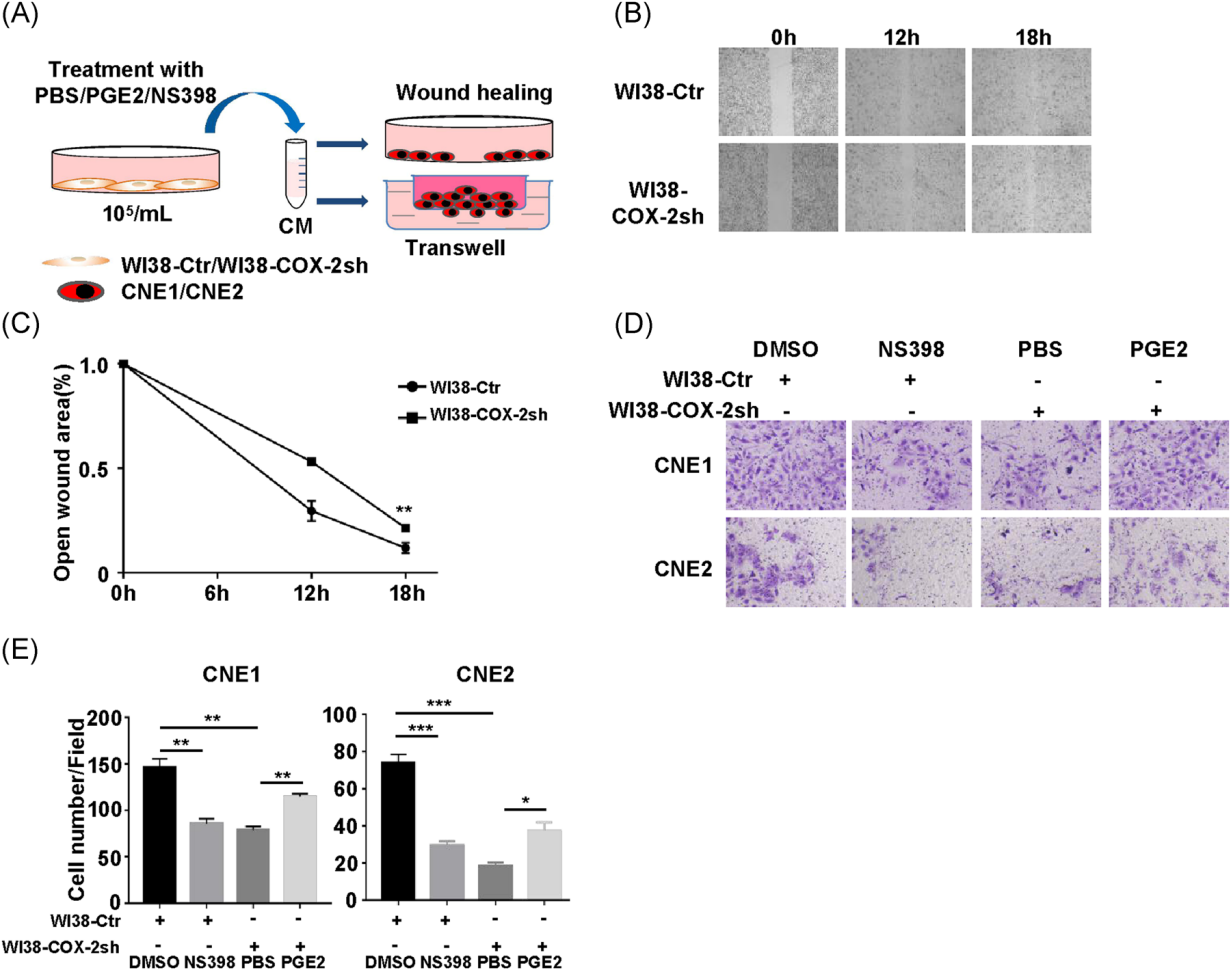
The COX‐2‐knockdown in WI38 cells attenuates migration and invasiveness of NPC cells. A, Schematic diagram for the treatment of NPC cells with CM collected from WI38 cell line. B, Representative images of the migration of CNE1 treated with WI38‐Ctr CM and WI38‐COX‐2sh CM at 0, 12, and 18 hours by the wound‐healing experiment. C, Open wound area analysis of CNE1 treated with WI38 CM at 18 hours. Bar, SEM. **P < .01 by unpaired *t* test. D, Representative images of the invasiveness of CNE1 and CNE2 treated with WI38 CM by transwell. E, Histograms represent the number of invaded cells. Bar, SEM. *P < .05, **P < .01, ***P < .001 by unpaired *t* test. CM, conditioned medium; COX‐2, cyclooxygenase‐2; NPC, nasopharyngeal carcinoma; SEM, standard error of the mean [Color figure can be viewed at wileyonlinelibrary.com]

Schematic diagram for the treatment of NPC cells with CM collected from WI38 cell line (Figure 4A). To further confirm that COX‐2 from fibroblasts affects the migration and invasiveness of NPC cells, another human lung fibroblast cell line WI38 was applied. We first constructed the WI38‐COX‐2sh cell line and also found CNE1 and CNE2 treated with CM from WI38‐COX‐2sh cells significantly has decreased migration (Figure 4B,C) and attenuated invasiveness phenotypes (Figure 4D,E). While PGE2 treatment reversed the inhibitory effect of CM from WI38‐COX‐2sh cells (Figure 4D,E), on the contrary, NS398 treatment reversed the enhanced effect of CM from WI38‐Ctr cells (Figure 4D,E). These results suggest that inhibition of COX‐2 in fibroblasts cells attenuates migration and invasiveness of NPC cells in vitro.

### 3.5 COX‐2 positively correlated with TNF‐α expression in CAF

**Figure 5:**
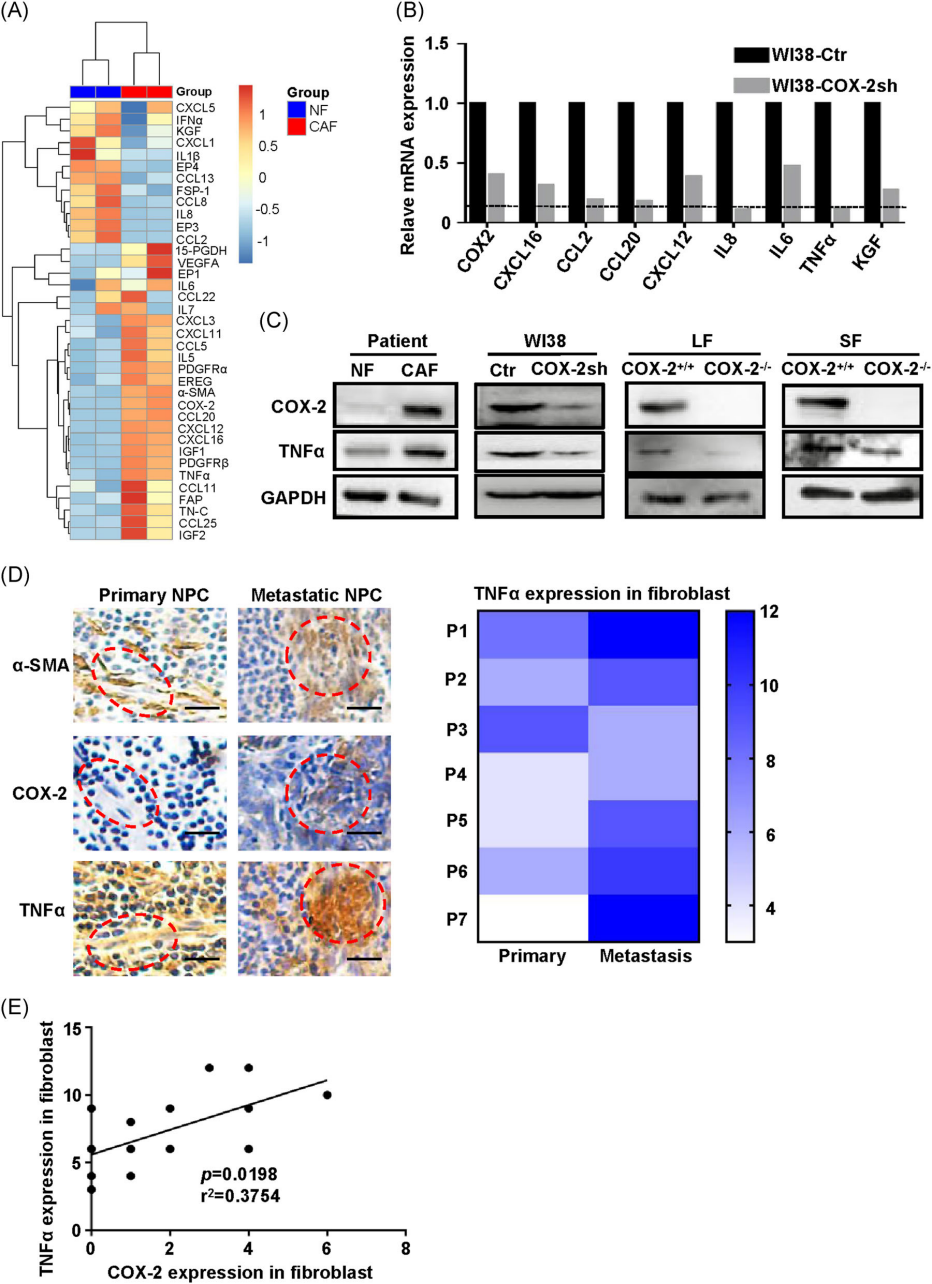
COX‐2 positively correlated with tumor necrosis factor‐α (TNF‐α) expression in CAF. A, Heatmap of COX‐2‐related genes, CAF markers, and inflammatory‐related genes were determined by qRT‐PCR array from paired NF and CAF. B, The mRNA expression of inflammatory‐related genes was detected by qRT‐PCR assay in WI38‐COX‐2sh cells. The genes were selected according to (A). C, The expression of COX‐2 and TNF‐α were determined by Western blot in patient fibroblast, WI38, LF and SF. Relative gradation corrected by glyceraldehyde 3‐phosphate dehydrogenase is shown below each band. D, Left, representative images of α‐SMA (top), COX‐2 (middle), and TNF‐α (bottom) IHC staining in paired NPC patients (n = 7) from primary and metastatic site. Scale bars, 20 μm. Right, Heatmap represent the TNF‐α score in fibroblast. *P < .05 by paired *t* test. E, The correlation analysis of COX‐2 and TNF‐α expression in fibroblast (n = 14). *P = .0198 by Correlation analysis. CAF, cancer‐associated fibroblast; COX‐2, cyclooxygenase‐2; IHC, immunohistochemistry; mRNA, messenger RNA; NF, normal fibroblast; NPC, nasopharyngeal carcinoma; qRT‐PCR, quantitative polymerase chain reaction; α‐SMA, α‐smooth muscle actin [Color figure can be viewed at wileyonlinelibrary.com]

Gene set enrichment analysis (GSEA) showed the inflammatory response was significantly enriched in CAF (Figure 1A), leading us to speculate that certain inflammatory cytokines secreted by CAF may be responsible for migration and invasiveness of NPC cells. We first examined the expression of 26 inflammatory cytokines, COX‐2‐related genes, and six CAF markers by using qRT‐PCR assay (Primer sequence; Table 2). Most inflammatory cytokines were upregulated in CAF compared with NF, including CXCL12, TNF‐α, and interleukin 6 (IL‐6; Figure 5A). Next, we investigated whether COX‐2 regulates the expression of those cytokines, we detected the expression of those cytokines in WI38‐COX‐2sh cells also by qRT‐PCR. Among these cytokines, CXCL12, TNF‐α, and IL‐6 were significantly reduced in the WI38‐COX‐2sh cells (Figure 5B).

Considering that TNF‐α is a signaling cytokine of NF‐κB pathway and COX‐2 acts as a target gene of NF‐κB signaling. We next investigated whether TNF‐α was indeed regulated by COX‐2. Enhanced TNF‐α expression was found in CAF compared with NF. Conversely, TNF‐α was downregulated either in WI38‐COX‐2sh cells or COX‐2^−/−^‐SF and COX‐2^−/−^‐LF (Figure 5C). Then, we assessed TNF‐α in seven paired NPC patients by IHC. Consistent with increased expression of COX‐2, the expression of TNF‐α was elevated in fibroblasts from metastatic NPC compared with primary NPC (Figure 5D). Interestingly, COX‐2 expression was found to significantly positively correlate with TNF‐α expression in seven paired NPC patients (Figure 5E). These results indicate that COX‐2 might be positively correlated with the expression of TNF‐α in NPC.

### 3.6 CAF promotes NPC cell migration and invasiveness through COX‐2–PGE2–TNF‐α axis

**Figure 6:**
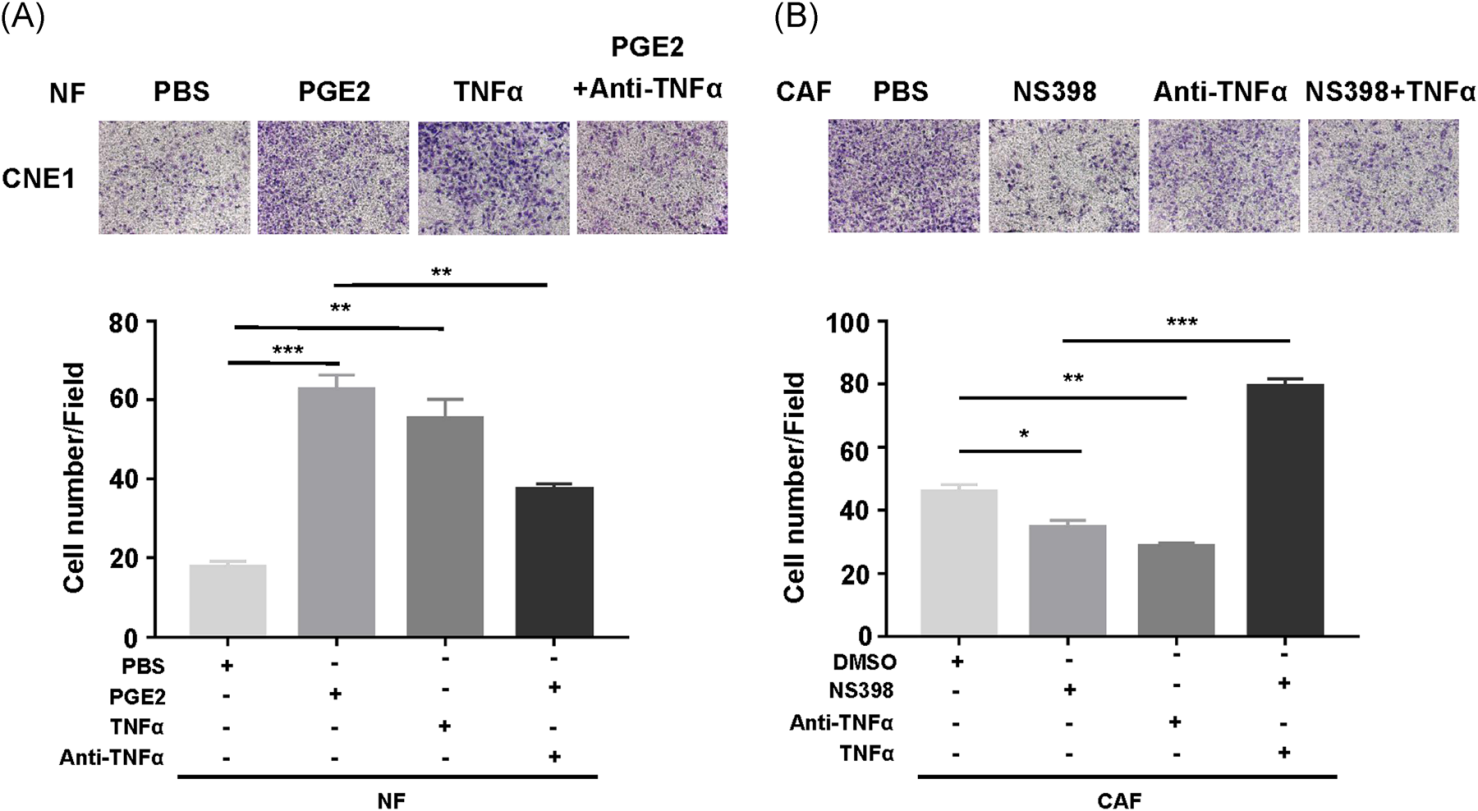
CAF promotes NPC cell migration and invasiveness through COX‐2–PGE2–TNF‐α axis. A, Representative images of the migration of CNE1 treated with CM from NF and CAF by transwell. PGE2+Anti‐TNF‐α CM shows NF+PGE2 was supplemented with anti‐TNF‐α (1:1000) before addition to the NPC cells, NS398+TNF‐α CM shows CAF+NS398 was supplemented with 10 ng/mL of TNF‐α. B, Histograms represent the number of migrate cells. Bar, SEM. *P < .05, **P < .01, ***P < .001 by unpaired *t* test. CAF, cancer‐associated fibroblast; COX‐2, cyclooxygenase‐2; CM, conditioned medium; NF, normal fibroblast; NPC, nasopharyngeal carcinoma; PGE2, prostaglandin E2; SEM, standard error of the mean; TNF‐α, tumor necrosis factor‐α [Color figure can be viewed at wileyonlinelibrary.com]

The previous study demonstrated that TNF‐α serves as a prognosis factor for NPC cells. However, whether and how COX‐2 induced TNF‐α expression in CAF to promote NPC metastasis is still unclear. To investigate whether COX‐2 promotes cell migration and invasiveness through TNF‐α, we examined the migration and invasiveness of CNE1 with treatment of TNF‐α recombinant protein or TNF‐α neutralizing antibody. As expected, TNF‐α rescued the inhibitory effect of CAF CM with NS398 on NPC cell metastasis, while TNF‐α neutralizing antibody reversed the enhanced effect of NF CM with high PGE2 (Figure 6A,B). We further assessed the impact of TNF‐α on CNE1 and CNE2 after the cells were exposed to CM from COX‐2^−/−^ LF and WI38‐COX‐2sh. Consistent with the results of the previous study, TNF‐α could also rescue the inhibitory effect of CM from COX‐2^−/−^ LF and WI38‐COX‐2sh (Figures S6A and S6B). Thus, these results suggested that COX‐2 in fibroblasts contributes to NPC cell metastasis through COX‐2‐PGE2‐TNF‐α axis (Figures S6A and S6B).

### 3.7 Host COX‐2 modulates lung metastasis of LLC cells correlated the expression of TNF‐α in vivo

**Figure 7:**
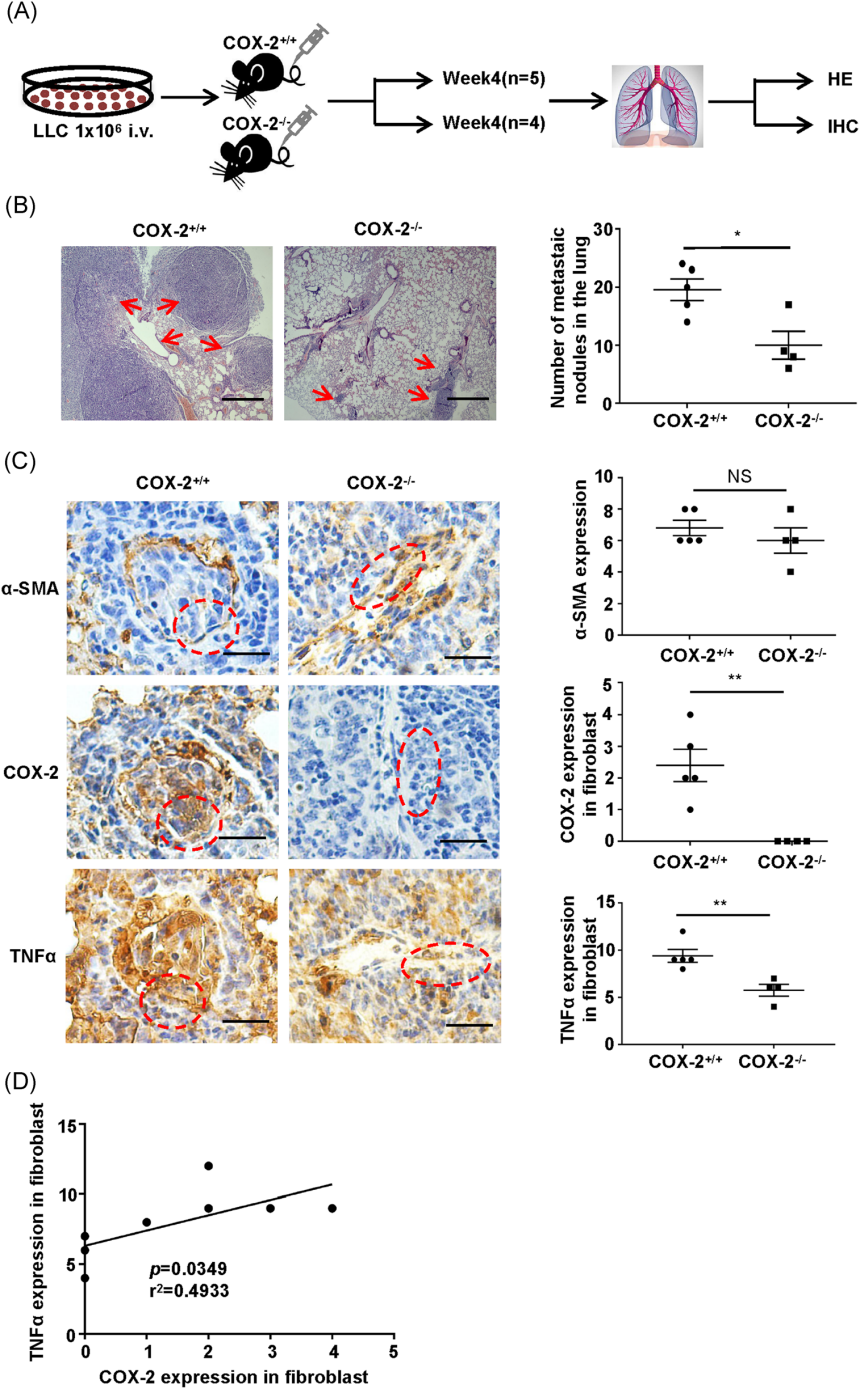
Host COX‐2 modulates lung metastasis of LLC cells correlated the expression of TNF‐α in vivo. A, A detailed delineation of the group distribution for in vivo experiment. B, Left, representative images of the lung metastatic nodules in COX‐2^+/+^ and COX‐2^−/−^ mouse by hematoxylin and eosin staining. Scale bars, 50 μm. Right, statistical chart represent the lung metastatic nodules in COX‐2^+/+^ and COX‐2^−/−^ mouse. Bar, SEM. *P < .05. C, Left, representative images for IHC detection of α‐SMA (top), COX‐2 (middle), and TNF‐α (bottom) protein in the lung section derived from COX‐2^+/+^ and COX‐2^−/−^ mouse injected with 10^6^ LLC cells intravenously. Scale bars, 20 μm. Right, statistical chart represent the α‐SMA, COX‐2, and TNF‐α score in fibroblast from lung section. Bar, SEM. NS, no significance, **P < .01. D, The correlation analysis of COX‐2 and TNF‐α expression in fibroblast (n = 9). *P = .0349 by correlation analysis. COX‐2, cyclooxygenase‐2; IHC, immunohistochemistry; SEM, standard error of the mean; TNF‐α, tumor necrosis factor‐α; α‐SMA, α‐smooth muscle actin [Color figure can be viewed at wileyonlinelibrary.com]

A detailed delineation of the group distribution for in vivo experiment (Figure 7A). To explore the COX‐2 function in vivo, LLC lung metastasis assay was applied in COX‐2^−/−^ mice. Briefly, COX‐2^+/+^ and COX‐2^−/−^ mice were injected with 10^6^ LLC cells intravenously. We found that COX‐2^+/+^ mice dramatically enhanced LLC metastasis to lung with increased metastatic nodules compared with COX‐2^−/−^ mice (Figure 7B). Then, we detected the COX‐2 and TNF‐α expression in the lung of fibroblasts, and we found that COX‐2 and TNF‐α are significantly high expressed in COX‐2^+/+^ mice (Figure 7C). Interestingly, COX‐2 expression was found significantly positively correlate with TNF‐α expression (Figure 7D). Taken together, these results showed that high COX‐2 in host fibroblasts affects lung metastasis of LLC cells.

## 4 DISCUSSION

Metastasis is the major cause of treatment failure in NPC and thus, preventing, predicting, and inhibiting metastasis is critical to improve treatment outcomes. In the current study, we reported the high expression of COX‐2 in CAF promotes NPC cells metastasis. Moreover, we observed that high COX‐2 expression in CAF was positively correlated with N stage, M stage, relapse, and survival in patients with NPC.

Previous studies reported that CAF markers include, but are not limited to, α‐SMA, FSP‐1, FAP, podoplanin (PDPN), PDGFR‐α/β, and NG2.^12^, ^18^ However, most of these markers are also expressed in other cell compartments and hence lack specificity for CAF/FSCs. For example, PDPN is also expressed in lymphatic endothelial cell.^36^, ^37^ NG2 and PDGFR‐β are commonly used to identify pericytes.^38^ In the stroma of pancreatic cancer, distinct populations of CAF differentially contribute to desmoplasia and inflammation and are molecularly distinguishable through α‐SMA expression and IL‐6 secretion.^39^ In this study, we found CAF derived from NPC represents the high expression of α‐SMA, FAP, PDGFR‐α/β, and TN‐C, consistent with other reports for the marker of CAF in other types of cancer.

COX‐2, an enzyme that catalyzes the formation of prostaglandins, affects tumor cell proliferation and host immune response and is undetectable in most of the normal tissue. Evidence from clinical and preclinical studies indicates that COX‐2‐derived prostaglandins participate in carcinogenesis, suppression of host immunity, apoptosis inhibition, angiogenesis, and tumor cell invasiveness and metastasis.^40^ Our previous studies showed that a high expression of COX‐2 is associated with the recurrence and a poor prognosis of patients with NPC, and COX‐2 may play a critical role in chemotherapeutic resistance in NPC via the inhibition of chemotherapy‐induced senescence via the inactivation of p53.^30^ However, these studies revealed that the COX‐2 expression in NPC cells, to the date, there is no report referred to the COX‐2 expression of TME in NPC. Several studies revealed that COX‐2 promotes tumor metastasis, for example, Zelei reported that COX‐2 is highly expressed in NPC cells, which promote the expansion of myeloid‐derived suppressor cells with a suppressive function on T cells through inducing the cytokine secretion including IL‐6 and GM‐CSF.^41^

In our study, we found that high expression of COX‐2 in CAF contributes to metastasis of NPC based on the following observations. First, at the clinical level, by applying clinical data and pathological sections of NPC (n = 43), we found that high COX‐2 expression in CAF was positively correlated with NPC metastasis. Interestingly, we obtained seven paired patients that primary and metastatic NPC tissues from the same patients, and we confirmed that low expression of COX‐2 in primary NPC tissues of fibroblast, but high expression of COX‐2 in metastatic site of CAF. Then, by applying migration and invasiveness assay, high expression of COX‐2 in CAF and PGE2 was produced and released from CAF facilitate metastasis in NPC in vitro. In this study, we demonstrated that COX‐2 induces PGE2 secretion in CAF and subsequently increases metastasis of NPC cells.

Our studies and other groups demonstrated that high expression of COX‐2 contributes to tumor cell proliferation, metastasis, and drug resistance through regulating several oncogenes or cell‐cycle‐related molecules such as p53, β‐catenin, Snail1, etc, in NPC and other cancers.^30^, ^41^, ^42^ However, in our study, we found TNF‐α, another new molecular positively correlated with COX‐2 by RNA‐Seq. Bourouba^43^ showed that TNF‐α promotes tumor growth via a NOS2‐dependent mechanism in NPC.^43^ This is the first time to demonstrate TNF‐α was upregulated in CAF in NPC. We detected TNF‐α expression in the paired NPC patients, and we found that high expression of TNF‐α and COX‐2 in metastatic site of CAF in NPC. Then, we found TNF‐α was decreased in COX‐2^−/−^ LF and WI38‐COX‐2sh and also has impaired invasiveness abilities in vitro. Finally, we employed a LLC lung metastasis assay in COX‐2^−/−^ mouse models, and we confirmed that the COX‐2 in host fibroblasts affect lung metastasis of LLC cells and correlated with the expression of TNF‐α in vivo. These results suggested that high expression of COX‐2 in fibroblasts promotes NPC metastasis through COX‐2–PGE2–TNF‐α axis. NS398 and anti‐TNF‐α significantly decreased the invasiveness abilities in vitro, and also suggested the potential therapeutic effect on CAF in NPC.

In summary, our study is the first to elucidate the critical role of COX‐2 in CAF in promoting NPC metastasis and predicting poor prognosis. Our results suggested that high expression of COX‐2 in CAF may serve as a new prognostic indicator for predicting NPC metastasis and provide the possibility of targeting CAF for treating advanced NPC.

## CONFLICT OF INTERESTS

The authors declare that there are no conflict of interests.

## Supporting information

Supporting information

Supporting information

Supporting information

Supporting information

Supporting information

## Data Availability

The datasets generated during and/or analyzed during the current study are available from the corresponding author on reasonable request.
