# Supplementary figures and images for: High COX‐2 expression in cancer‐associated fibiroblasts contributes to poor survival and promotes migration and invasiveness in nasopharyngeal carcinoma
Source: Mol Carcinog. 2019 Dec 22;59(3):265–80. doi: 10.1002/mc.23150 (PMC7027878; doi:10.1002/mc.23150)

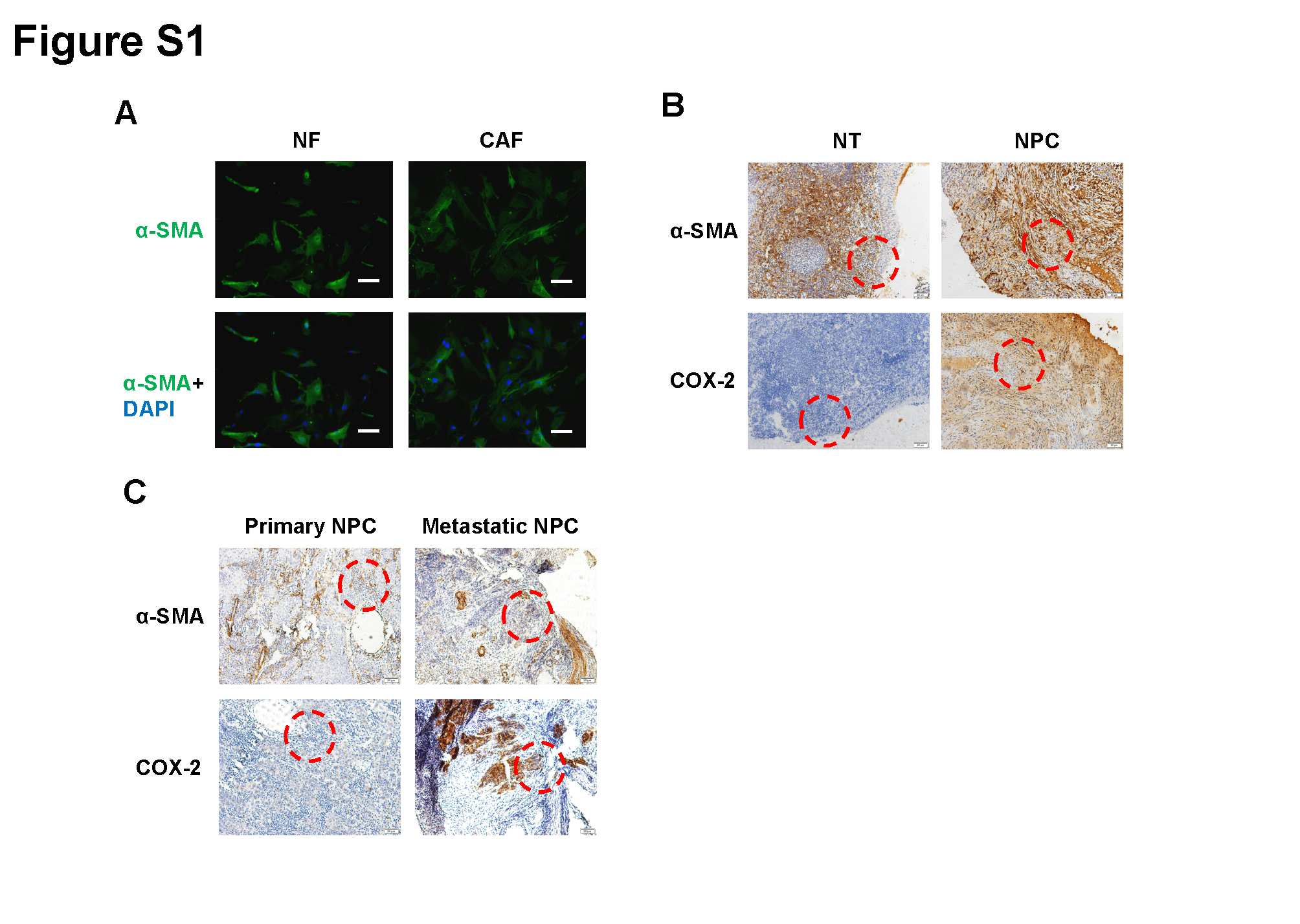

Supplement: Supplementary file 1 — Supporting information [file MC-59-265-s001.tif]

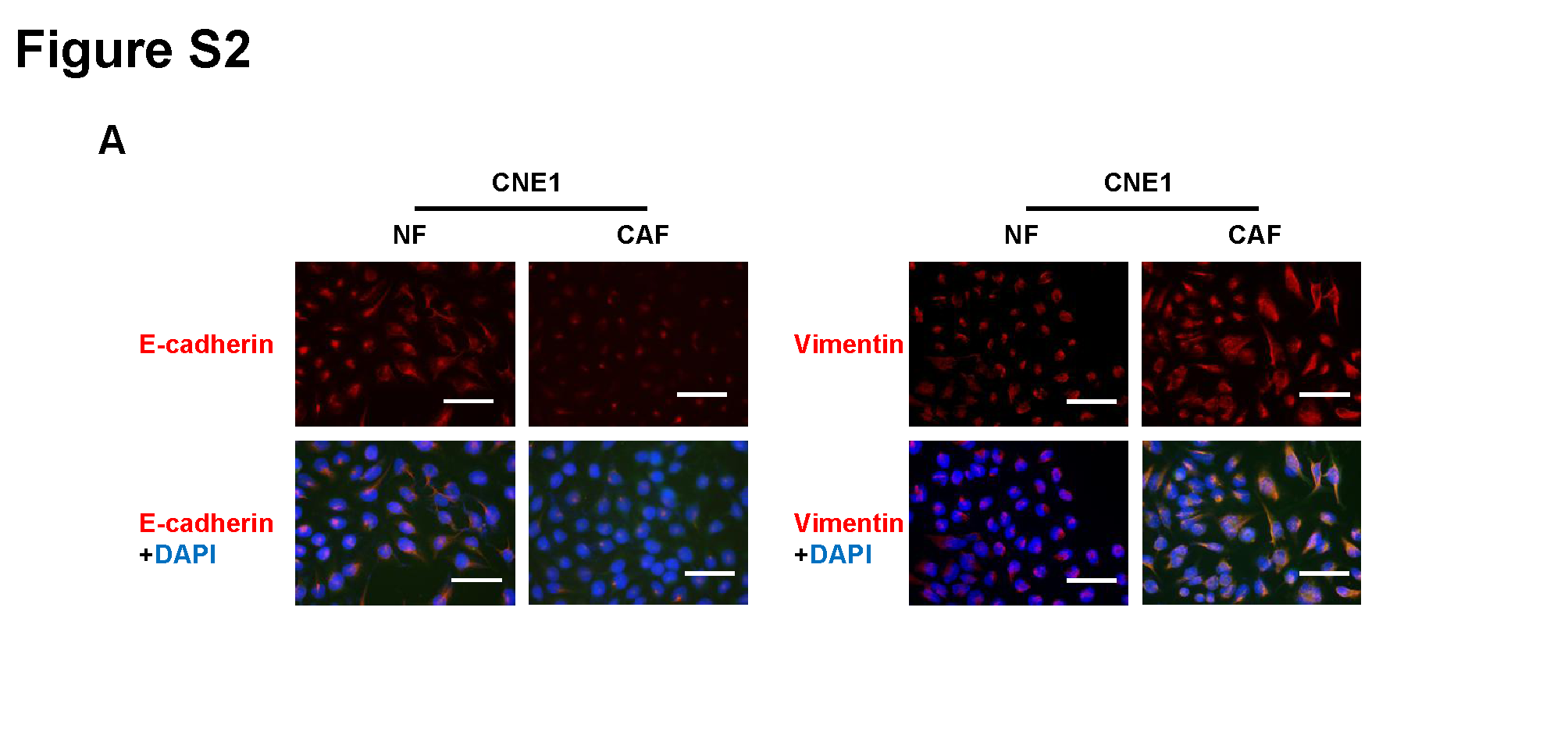

Supplement: Supplementary file 2 — Supporting information [file MC-59-265-s002.tif]

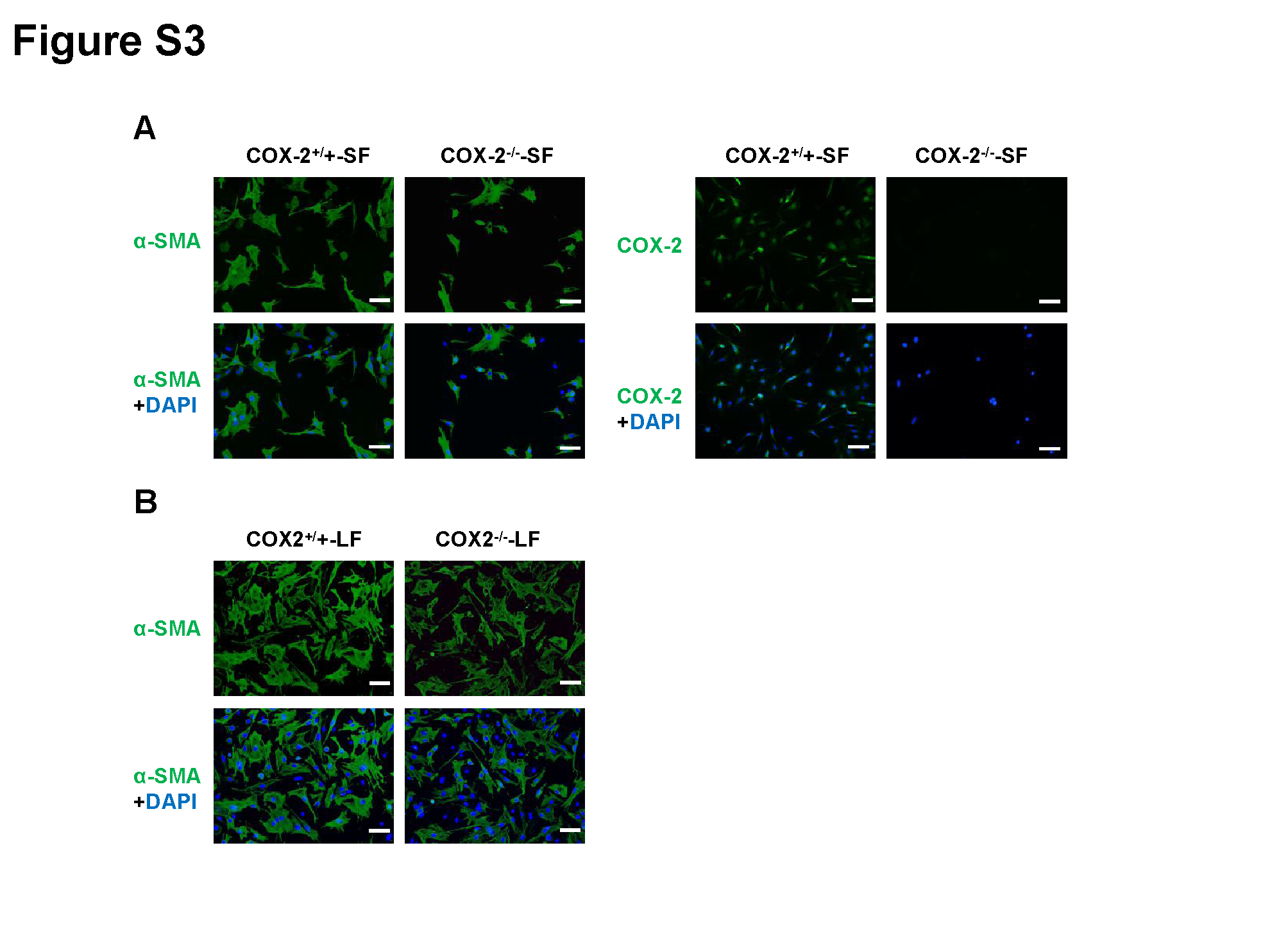

Supplement: Supplementary file 3 — Supporting information [file MC-59-265-s003.tif]

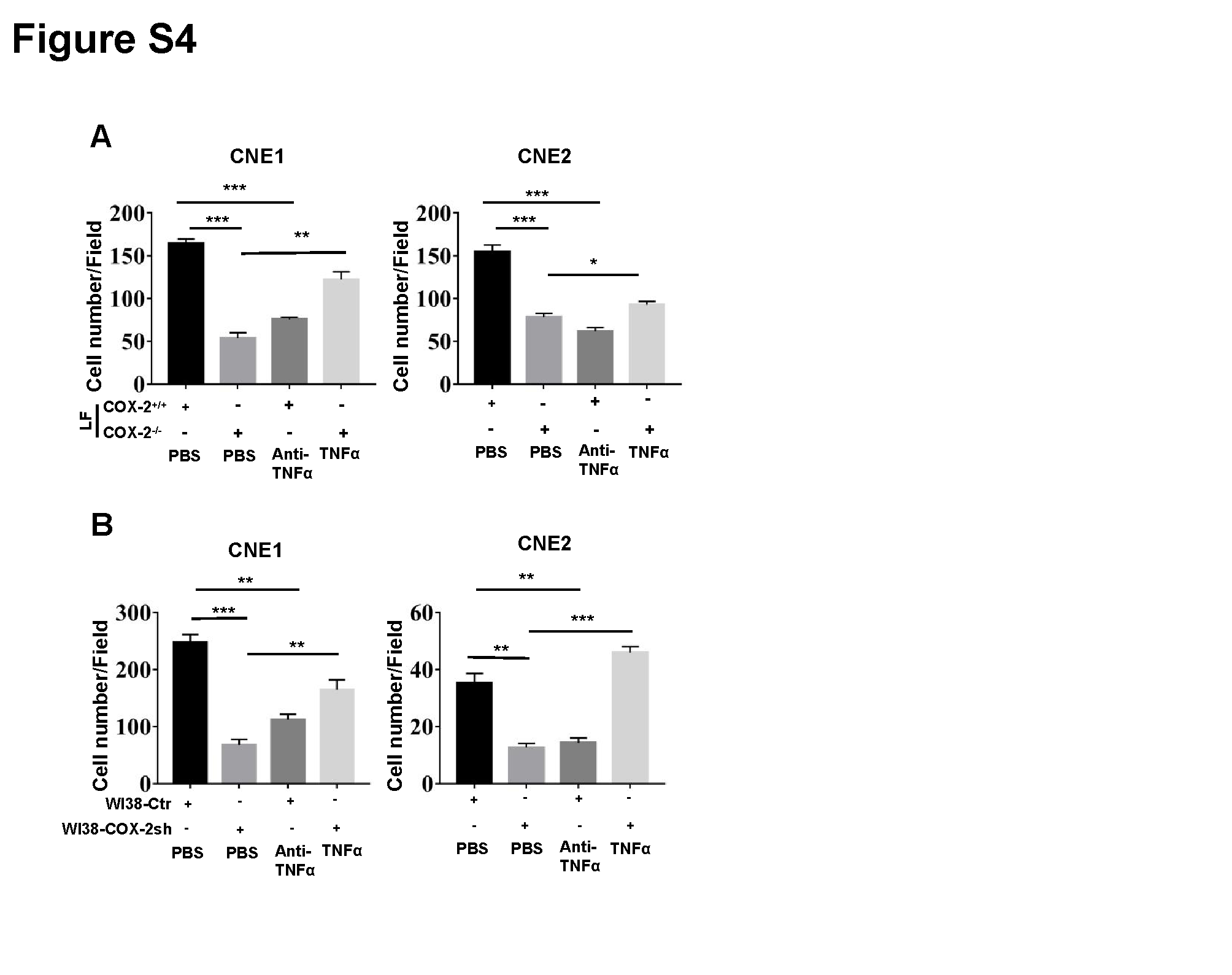

Supplement: Supplementary file 4 — Supporting information [file MC-59-265-s004.tif]
