# Supplementary material for: High COX‐2 expression in cancer‐associated fibiroblasts contributes to poor survival and promotes migration and invasiveness in nasopharyngeal carcinoma
Source: Mol Carcinog. 2019 Dec 22;59(3):265–80. doi: 10.1002/mc.23150 (PMC7027878; doi:10.1002/mc.23150)
Supplement: Supplementary file 5 — Supporting information [file MC-59-265-s005.doc]

**Legends to Figures**

**Figure S1, related to Figure 1. COX-2 expression was up-regulated in CAF and correlates with metastasis in NPC**

1. Representative images of α-SMA expression in NF and CAF by IF. DAPI, blue; α-SMA, green. Scale bars, 10μm.
2. Representative images of α-SMA and COX-2 IHC staining in NT and NPC from Fig. 1D. Scale bars, 50μm.
3. Representative images of α-SMA and COX-2 IHC staining in paired patients at primary site and distant metastasis site from Fig. 1E. Scale bars, 50μm.

**Figure S2, related to Figure 2. Increased Vimentin and decreased E-cadherin expression in NPC cells treated by CM from CAF**

(A)Left, representative images of E-cadherin expression in CNE1 treated with CM from NF and CAF by IF. DAPI, blue; E-cadherin, red. Scale bars, 10μm. Right, representative images of Vimentin expression in CNE1 treated with CM from NF and CAF by IF. DAPI, blue; Vimentin, red. Scale bars, 10μm.

**Figure S3, related to Figure 3. The expression of α-SMA and COX-2 in skin and lung fibroblasts generated from COX-2 knockout mice**

1. Representative images of α-SMA and COX-2 expression in COX-2^+/+^-SF and COX-2^-/-^-SF by IF. DAPI, blue; α-SMA and COX-2, green. Scale bars, 10μm.
2. Representative images of α-SMA expression in COX-2^+/+^-LF and COX-2^-/-^-LF by IF. DAPI, blue; α-SMA, green. Scale bars, 10μm.

**Figure S4, related to Figure 6. CAF promotes NPC cell migration and invasiveness through COX-2-PGE2-TNFα axis**

1. Histograms represent the number of migrate cells treated with LF CM. Bar, SEM. *, *p*<0.05, **, *p*<0.01, ***, *p*<0.001 by Unpaired t test.
2. Histograms represent the number of migrate cells treated with WI38 CM. Bar, SEM. *, **, *p*<0.01, ***, *p*<0.001 by Unpaired t test.
